# Simulation and experimental study of calcium sulfate and barium sulfate scale formation and Inhibition in petroleum engineering

**DOI:** 10.1038/s41598-025-33276-0

**Published:** 2025-12-20

**Authors:** Zahra Zare, Leila Mahmoodi, M. Reza Malayeri

**Affiliations:** Department of Chemical Engineering, School of Chemical and Petroleum Engineering, Shiraz University, Shiraz, Iran

**Keywords:** Barium sulfate, Calcium sulfate, Folic acid, Inhibition, Simulation, Scaling, Chemistry, Environmental sciences, Materials science

## Abstract

Petroleum engineering could engage numerous challenges caused by sulfate mineral precipitation and deposition. This severe critical issue which could directly lead to production reduction, was investigated in this study. Accordingly, the precipitation, deposition, and inhibition of calcium and barium sulfate were scrutinized from the simulation and experimental perspectives. The simulation tools, PHREEQC and Aspen Plus, were first used to corroborate the results of the high-temperature (90 °C) standard static experimental tests conducted for calcium and barium sulfate precipitation and deposition phenomena. In the experimental phase of the inhibition study, folic acid was evaluated as a green scale inhibitor (SI) and compared to a phosphonate-based commercial SI regarding inhibition efficiency (IE%) and inhibition mechanisms. The findings indicated that folic acid reduced calcium and barium sulfate precipitation as much as 50.8% and 44.8% respectively at the specific critical mixing ratios through the crystal modification inhibition mechanism provided by scanning electron microscopy (SEM) analysis. Moreover, folic acid could perform effectively in the mitigation of calcium and barium deposition by 53.7% and 47.2% in the presence of 5 g of dolomite rock. However, a comparison of a commercial SI and folic acid showed that the commercial SI was weaker than folic acid for mitigation of sulfate deposition through the threshold inhibition mechanism.

## Introduction

 The petroleum industry as one of the most vital sources of energy generally faces numerous challenges in oilfield production and exploitation. One of the most costly and severe challenges is the mineral scales of carbonate and sulfate^1,2^. Inorganic scale would cause permeability reduction, the existing deposits pipes and equipment clogging, operational and maintenance costs increasing, and ultimately production efficiency reduction^3–7^. Calcium, barium, and strontium sulfate are known as the pH-independent sulfate scales^6,8^ which are not acid-washable and normally require other treatments to mitigate^9^.

Sulfate scales are commonly exacerbated in water injection operations for enhanced oil recovery due to the chemical incompatibility between the injection and formation water based on the differences in the ionic composition of the aforementioned waters^8,10^. The formation water is usually supersaturated in cations such as barium $\:{(Ba}^{2+})$, strontium $\:{(Sr}^{2+})$, and calcium ($\:{Ca}^{2+}$), while the injection water dominantly contains the anions of sulfate ($\:{SO}_{4}^{2-}$)^5,6,11,12^. Therefore, the precipitation of the inorganic sulfate particles would be expected in the common operating conditions in almost all stages of oil production, as the result of high temperatures and complex fluid chemistry of incompatible mixing waters.

Accordingly, the high demand for dealing with this intense issue would be predicted. The mechanical and chemical techniques are typically utilized to overcome the sulfate precipitation and deposition, respectively^6,8,9,13^. Although mechanical methods are effective for removing the existing deposits from well columns, chemical methods are often preferred due to the high costs and potential for formation damage associated with mechanical methods^8,14,15^. On the other hand, among the chemical methods, utilization of the substances named scale inhibitors (SI) are well-known to mitigate scale formation^9,16,17^ by the various mechanisms of threshold inhibition and crystal modification^18–20^.

The most common types of SIs, including (i) inorganic phosphates, i.e., polyphosphates, (ii) organic phosphorus, i.e., organophosphorus, and (iii) organic polymers, have been developed with extensive properties and applications^9^. Consequently, several studies have focused on the performance of different SIs exposed to the sulfate precipitated particles at different experimental conditions through standard static tests.

For instance, acrylate and methacrylate polymers^21^, phosphonobutane tricarboxylic acid (PBTC)^22,23^, phosphinopolycarboxylic acid (PPCA), sulfonated polycarboxylic acid (SPCA)^24^, and hexamethylenediamine tetra (HDTMP)^25^ were experimentally investigated. The results indicated that polyacrylate efficiency was determined by polymer charge density and molecular weight strongly, while PBTC, SPCA, and PPCA significantly inhibited scaling rates at high temperatures of up to 150 °C, although their efficiency decreased with increase in temperature. HDTMP inhibited strongly under low saturation index conditions and at temperatures up to 100 °C. Aminopolycarboxylate compounds such as diethylene triamine penta acid (DTPA) and triamine penta acid (TPA)^26^ were also evaluated at extremely low levels.

These inhibitors worked well to inhibit barium sulfate scaling at as low as 0.5–2 mg/L concentrations, with performance influenced by solution composition and ionic strength. Hydroxy ethylenediphosphonic acid (HEDP) and phosphonobutanetricarboxylic acid (PBTC)^26^ were evaluated using thermodynamic modeling. HEDP worked better at lower dosages and in undersaturated solutions, while PBTC worked better at high dosages and in high scaling tendency solutions. Benzotriazoletriphosphonic acid (BTTPA) and triazine triphosphonate (TTP)^27^ were investigated for their effects on the nucleation and crystal growth of calcium sulfate. BTTPA suppressed nucleation adequately and altered crystal morphology, while TPA suppressed moderately, particularly in near-neutral pH conditions.

However, environmental regulations require the utilization of environmentally friendly scale inhibitors which should meet three criteria of (i) biodegradability, (ii) no-bioaccumulation, and (iii) non-toxicity^28^, despite the commercial inhibitors demonstrate reasonable effectiveness. Accordingly, recent researches have been directed toward development and utilization of environmentally friendly SIs. For instance, Helianthus annuus^29^ as a common sunflower, lignosulfo nates^30^, salicylic acid^31^, persimmon leaf^32^, natural polysaccharides like carboxymethyl cellulose (CMC), carboxymethyl starch (CMS), chitosan^33^, inulin, vitamin B, and folic acid^34–36^ could be named as the newly tested green SIs under various experimental conditions of temperature, mixing ratio, and the presence of the rock surface for both mineral precipitation and deposition phenomena. Sunflower seed extract showed complete inhibition (100%) of precipitation of calcium sulfate and 84% for barium sulfate at room temperature with excellent morphological modification in crystals when compared to synthetic phosphonates^29^. Folic acid and inulin performed optimally at with inhibition efficiency enhanced with a rise in temperature up to 65% and 54% inhibition of precipitation at 90 °C^34–36^. In the static jar tests at elevated temperatures, CMC was more effective than CMS and chitosan in inhibiting calcium sulfate at 90–95 °C^33^. Salicylic acid showed high efficiency (up to 98.9%) at 6 mg/L under pH-dependent conditions in the static tests, but its efficiency dropped under higher temperature and supersaturation conditions^31^. Under dynamic flow conditions, folic acid reached up to 49% scale inhibition in dolomite cores, while inulin reached 39% in sandstone, implying the involvement of surface mineralogy in scale deposition^36^. Surface energy calculations and SEM investigations confirmed that folic acid works mainly through crystal modification, while inulin works through a threshold inhibition mechanism. Additionally, in incompatible water systems the mixing ratio had a great effect on scale inhibition, where green scale inhibitors had positive effects in inhibiting scale even under brine-brine and brine-rock interactions interaction conditions^34^.

Given the focus on environmentally friendly inhibitors, nonetheless the existing studies have mainly directed towards carbonate precipitation and/or deposition. Therefore, a research gap exists with respect to investigation of tough sulfate particles, particularly under high-temperature conditions. Therefore, this study aims to evaluate the performance of an environmentally friendly SI, folic acid, overcoming the calcium and barium sulfate precipitation and deposition in the absence and presence of the dolomite rock surface, respectively. In this regard, standard static test at 90 °C was applied to scrutinize the inhibition performance of various concentrations of folic acid in an incompatible solution formed in the case of the anionic and cationic solution at the specified critical mixing ratio. Additionally, scanning electron microscopy (SEM) and energy dispersive X-ray (EDX) analyses were performed to identify the inhibition mechanism through morphology and elemental composition of the collected precipitations.

The optimal concentration leading to the minimum amount of precipitation was employed in the deposition high-temperature experiments containing the incompatible mixing solutions at the critical mixing ratio exposed to 5 g of dolomite rock to further assess the green SI potential in deposition phenomena. A phosphonate-based commercial SI was also compared with folic acid in all steps of the experiments. It should finally be mentioned that in this study, PHREEQC and Aspen Plus simulation tools were used to address two matters of (i) determining the critical mixing ratio (leading to the maximum amount of precipitation) which would be indispensable for doing the follow-up experiments, and (ii) validating the sulfate precipitation/deposition experimental results.

## Materials and experimental procedure

### Materials

#### Solutions and scale inhibitor

Two various cationic solutions were prepared by dissolving precisely defined amounts of high-purity calcium chloride dihydrate (*CaCl*_*2*_*·2H*_*2*_*O*), and barium chloride dihydrate (*BaCl*_*2*_*·2H*_*2*_*O*) salts in deionized water. Furthermore, the anionic solution was individually prepared by dissolving high-purity sodium sulfate salt (*Na*_*2*_*SO*_*4*_) in deionized water at the ambient temperature and pressure.

It is worth mentioning that the cationic and anionic solutions investigated in this study were modeled after the North Sea water composition, which had been previously scrutinized in the literature. This choice was made based on the assertion that the nature of the regional oil-bearing formations makes this area more susceptible to sulfate scale formation due to the potential incompatibility with seawater^37^. The considered concentration of various ions in every solution, as well as the required mass of the appropriate salts to distinctly prepare each solution, are presented in Table 1.

**Table 1:** Ionic concentration of the studied solutions.

Solution	Cationic			Anionic		
Ion	Ion concentration (mg/L)	Salt used	Mass of salt (g/L)	Ion concentration (mg/L)	Salt used	Mass of salt (g/L)
*Ca* ^*2+*^	2000	*CaCl*_*2*_ *0.2 H*_*2*_ *O*	7.723	-	-	-
*Ba* ^*2+*^	296	*BaCl*_*2*_ *0.2 H*_*2*_ *O*	0.504	-	-	-
$\:{SO}_{4}^{2-}$	**-**	-		2960	*Na*_*2*_ *SO*_*4*_	4.376

A green scale inhibitor was experimentally scrutinized in this work. For this purpose, three different concentrations (500, 1000, and 2000 mg/L) of folic acid were evaluated in the incompatible mixing of the solutions. It is worthwhile to note that the previous studies have investigated the efficacy of folic acid at lower concentration ranges^34–36^. In the present study though, a higher concentration range was selected to identify the dosage required to achieve a considerable inhibition efficiency under more severe sulfate precipitation conditions, as compared to carbonate scales. This approach helped to provide a more comprehensive understanding of the potential of this green scale inhibitor under varying scaling scenarios.

It was proposed to assess its potential to abate the sulfate particles’ precipitation, particularly through the carboxylic acid (-COOH) groups in its chemical structure^34,35^. However, a commercial phosphonate-based scale inhibitor was further examined to compare with folic acid performance in order to answer the question of whether folic acid could be an alternative or not.

## Rock

Crushed Dolomite rock was used to evaluate the interactions between deposited particles and scale inhibitors on the rock surface. The X-ray diffraction (XRD) results showed that the mineral composition of the rock sample included 94% dolomite^36^ to ensure pure dolomite rock surface in presenting the experimental findings as well as PHREEQC simulations which will be explained in the following (Sects. "PHREEQC").

### Experimental procedure

Standard static tests are commonly employed to examine the performance of SI at high temperatures^34,35,38–41^. For this purpose, the cationic and anionic solutions were first prepared according to the ion concentration presented in Table 1. Afterward, the incompatible cationic and anionic solutions, which included one type of cation and anion, were mixed at a specified volume ratio (volume percentage, %). The mixing could provide an environment to precipitate calcium sulfate and barium sulfate according to the chemical reactions stated in Eqs. 1 & 2, respectively.

1 $$\:{Ca}^{2+}\:+\:\:{SO}_{4}^{2-}\:\:\to\:{CaSO}_{4}\downarrow\:$$

2 $$\:{Ba}^{2+}\:+\:\:{SO}_{4}^{2-}\:\to\:{BaSO}_{4}\downarrow\:\:$$

It should be pointed out that, in addition to the solution without any SI (blank solution), three various folic acid concentrations (500, 1000, and 2000 mg/L) were added to the anionic solution before incompatible mixing of the investigated solutions in a certain mixing ratio of cationic/anionic.

Next, the mixed samples were kept at 90 °C for 7 days to ensure that the system reached equilibrium and this period is sufficient for mineral scale growth^34,35,39^. After seven days, the samples were immediately filtered using cellulose nitrate filters with a pore diameter of 0.20 microns to collect the precipitated particles for further analysis of SEM/EDX as well as inhibition efficiency calculation based on the mass changes of precipitated/deposited particles^42^ expressing in Eq. (3):

3 $$\:\%IE\:\left(MASS\right)=\frac{{M}_{B}-\:{M}_{S}}{{M}_{B}}\:\times\:100$$

where *M*_*B*_ and *M*_*S*_ are the mass of the precipitate/deposited particles in the absence and presence of an SI, respectively^42^.

It is worth mentioning that the ion concentration of the filtered samples was measured using Atomic Absorption Spectroscopy (AAS) to determine the metal cation concentrations that were requisite in inhibition efficiency (%IE) calculation. It was done by Eq. (4) according to the NACE standard^38^ where [$\:{M}^{2+}$] showed any investigated divalent cation.

4 $$\:\%IE\:\left(NACE\right)=\frac{\:{{[M}^{2+}]}_{Sample}-\:{{[M}^{2+}]}_{Blank}}{\:\:{{[M}^{2+}]}_{initial}-\:{{[M}^{2+}]}_{Blank}}\:\times\:100$$

By the use of Eq. (4), it is also noteworthy that $\:{{[M}^{2+}]}_{Sample}$ and $\:{{[M}^{2+}]}_{Blank}\:$are the concentration of the cation in the sample in the presence and absence of SI, respectively, and $\:{{[M}^{2+}]}_{initial}$ is the initial concentration of the cation immediately after the brine solutions were mixed, before heating^38^. To ensure the reliability and consistency of the experimental results, each experiment was repeated three times to obtain the results with an error of ± 5%. Although, the calculated inhibition efficiencies are basically different and the effect of ion re-adsorption on the filter and dolomite surfaces would not be clarified, the research concentrate on the similar trend of estimated %IE versus SI’s concentrations.

**Fig. 1:**
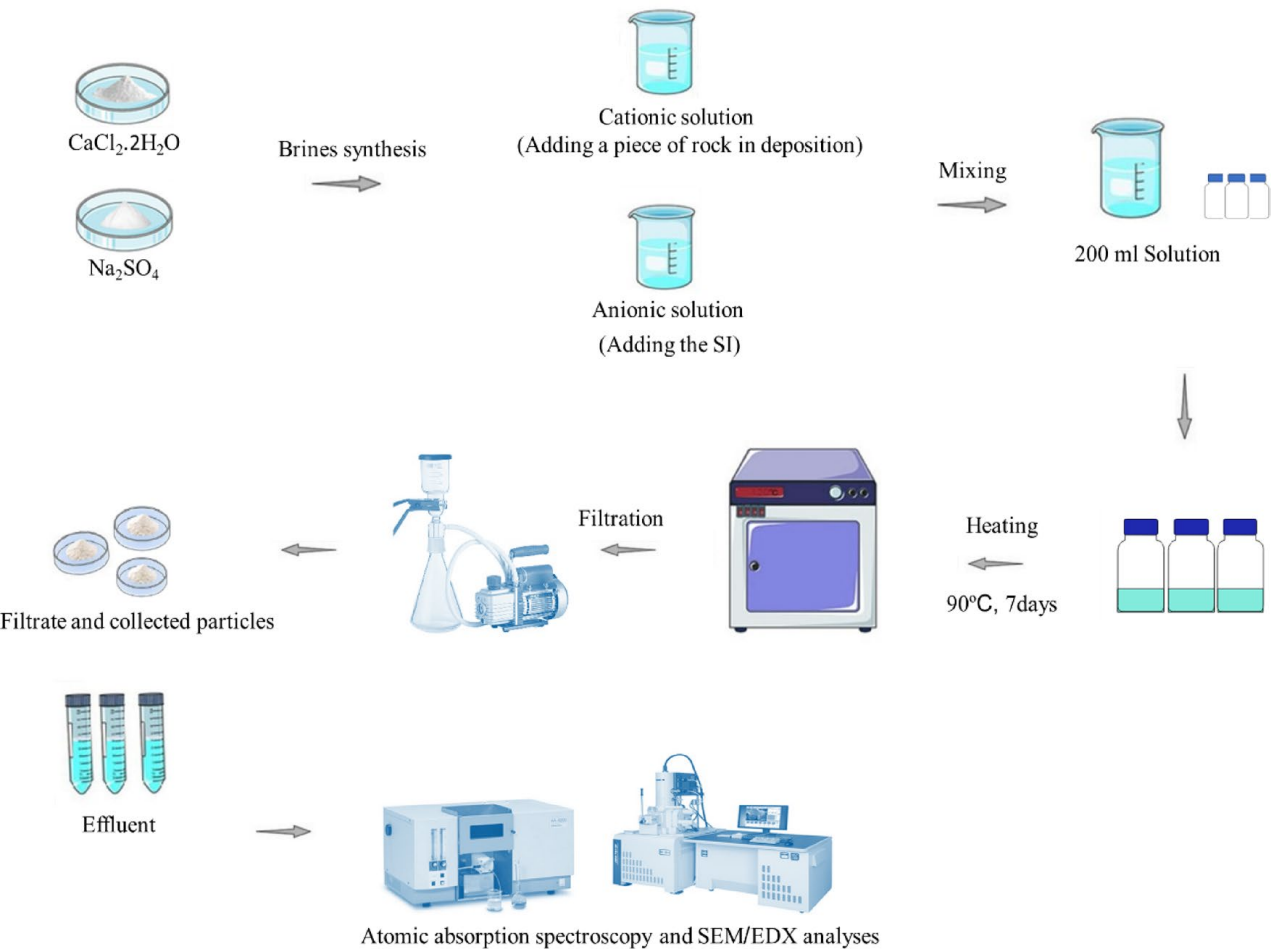
Schematic of applied experimental procure in this study.

The experimental conditions and schematic of the standard static tests are eventually presented in Table 2; Fig. 1, respectively. However, one point should be noted about the difference in the critical mixing ratio of calcium sulfate and barium sulfate investigation. The definite difference mixing ratios were the direct results of the simulation study by PHREEQC which will be more explained in Sects. 4 − 1.

**Table 2:** High temperature static test conditions.

Parameter	Value/range
Temperature (ºC)	90
Pressure (atm)	1
Total volume (L)	0.2
Heating time (day)	7
Mixing ratio of cationic/anionic (%)	40:60 (for *CaSO*_*4*_) 90:10 (for *BaSO*_*4*_)
Folic acid concentration (mg/L)	500, 1000, and 2000
Commercial SI concentration (mL/L)	15
Dolomite rock (gr)	5

In addition, the efficiency of the SI was also scrutinized in the presence of the dolomite rock surface separately. The only difference in the experimental procedure between the precipitation and deposition phenomena was the presence of the rock surface. In addition to initially placing a certain amount of piece of dolomite rock (5 gr) to the cationic solution (without stirring during the experimental period), all of the above steps were repeated.

### **Simulation study**

Advanced simulation tools of PHREEQC and Aspen Plus were employed in this study to quantitatively predict the amount of precipitated/deposited sulfate particles under the investigated conditions. Notably, neither software included scale inhibitors, allowing for a straightforward evaluation of scale inhibitor performance and its broader system-level impacts.

By integrating results from both PHREEQC and Aspen Plus, the study would provide an extensive validation of the experimental results of sulfate particles precipitation and deposition in the absence of scale inhibitors. Notably, both simulation tools of PHREEQC and Aspen Plus are based on chemical reactions presented by Eqs. 1 and 2 which showed that the molar ratio of cation to anion was equal to 1:1.

The following sections detail the methodologies and assumptions of these simulations, emphasizing the critical conditions for the particles’ precipitation and management.

### PHREEQC

PHREEQC, originally developed by the U.S. Geological Survey, is a powerful geochemical modeling software specifically designed for simulating chemical reactions and equilibria in aqueous systems^35,43–48^. Its capabilities include speciation modeling, saturation index (SI) calculations, surface complexation modeling, ion exchange, and kinetic reactions, making it particularly suitable for evaluating scale formation and mineral interactions in complex reservoir and water flood scenarios.

In this study, PHREEQC was employed to conduct a comprehensive analysis of the precipitation and deposition (in the presence of the dolomite rock surface) behavior of sulfate particles of calcium and barium sulfates, under various mixing conditions. The simulations were performed in the absence of any scale inhibitors to intrinsically investigate the scaling potential of the system. Aqueous solutions containing incompatible cationic ($\:{Ca}^{2+}$, $\:{Ba}^{2+})$ and anionic ($\:{SO}_{4}^{2-})\:$species were mixed at different mixing ratios and temperatures while maintaining atmospheric pressure (1 atm). The software enabled the calculation of saturation indices, ion speciation, and the extent of precipitation under each condition, allowing for the identification of critical mixing ratios that promoted maximum precipitation.

Moreover, by defining dolomite as a reactive surface within PHREEQC, it was allowed to simulate the deposition process to investigate the influence pf the rock presence on the mass of deposition.

The ultimate objective of this simulation was to understand how varying operational parameters such as temperature, mixing ratio, and presence of dolomite rock surfaces could govern sulfate scale precipitation and deposition. The PHREEQC results (Sect. "Simulated results: PHREEQC") will be systematically compare with experimental observations, and would serve as a validation tool, enhancing the confidence in both the simulation methodology and the experimental data.

### Aspen plus

Aspen Plus is a comprehensive process simulation software widely used for modeling and analyzing complex chemical systems. In this study, hence, it was utilized to simulate the precipitation of the calcium and barium sulfate particles in the absence of scale inhibitors through a crystallization process.

The simulation was conducted under isothermal and isobaric conditions (T = 90 °C, *P* = 1 atm) with no pressure drops along the process stream. Consequently, the Aspen Plus methodology relied solely on stoichiometric reactions and internal databanks to predict equilibrium, without incorporating external kinetic constants. The simulation setup incorporated experimental flow rates of the cationic and anionic solutions and was configured to run continuously for seven days, mimicking the temporal scale of the laboratory experiments. The crystallizer block was employed as the core unit to model the nucleation and growth of mineral solid particles, wherein *CaCl*_*2*_ and *BaCl*_*2*_ were defined as limiting reactants to realistically reflect the depletion behavior observed in practice.

**Fig. 2:**
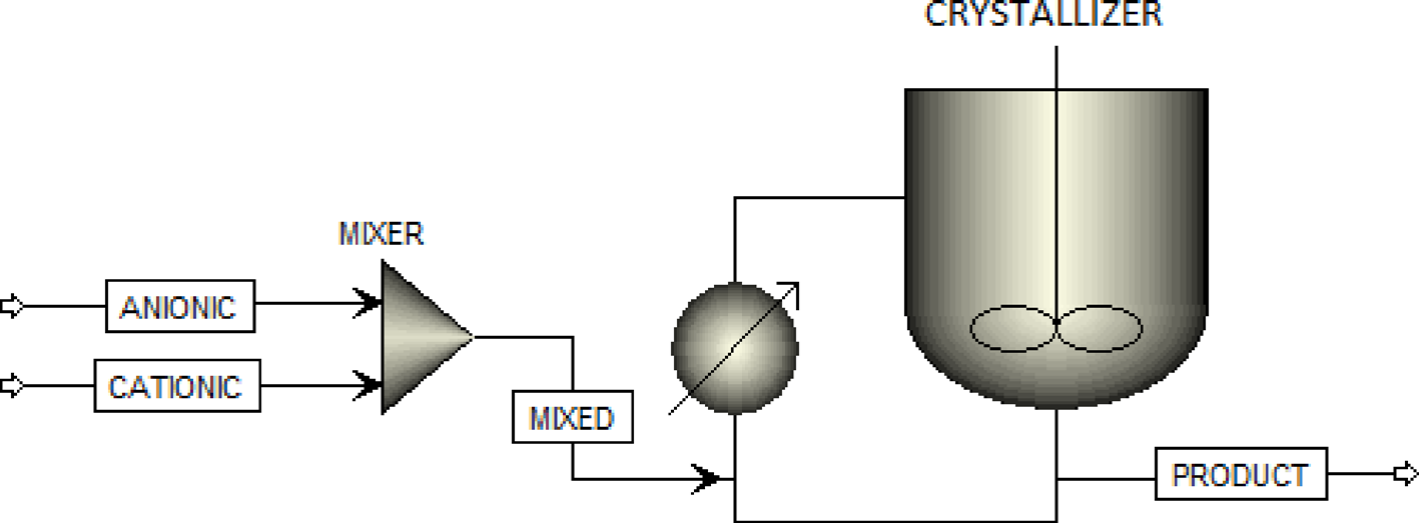
Schematic of Aspen plus simulator.

Figure 2 illustrates the process flow diagram constructed within Aspen Plus, which mirrors the operational sequence used in the experimental setup. Through this approach, Aspen Plus enabled the prediction of the extent of precipitation over a certain period. Importantly, the simulation results (Sects. "Simulated results: Aspen Plus") were also used to validate and cross-check the experimental observations as well as the PHREEQC results, thereby enhancing the overall reliability and consistency of the findings, as the main purpose.

It is well established that PHREEQC and Aspen Plus simulate precipitation processes from thermodynamic and kinetic perspectives, respectively. In this study, PHREEQC was employed to predict the equilibrium conditions favoring sulfate scale formation based on ionic speciation and saturation indices. Concurrently, Aspen Plus was used to simulate the process flow and kinetically assess the precipitation under dynamic operational scenarios over a period of 7 days. This simulation effort aimed not only to cross-validate the experimental findings but also to compare the precipitation potency of calcium and barium sulfate. The results obtained from the Aspen Plus simulations will be discussed in detail in Sect. "Simulated results: Aspen plus".

## Results and discussion

### Simulated results: PHREEQC

In the present work, the impact of two parameters of the mixing ratio of cationic/anionic solutions and temperature on the mass of precipitated and also deposited inorganic particles of calcium sulfate (gypsum) and barium sulfate (barite) have been investigated by the use of PHREEQC simulation tool.

### Impact of mixing ratio on precipitation

**Fig. 3:**
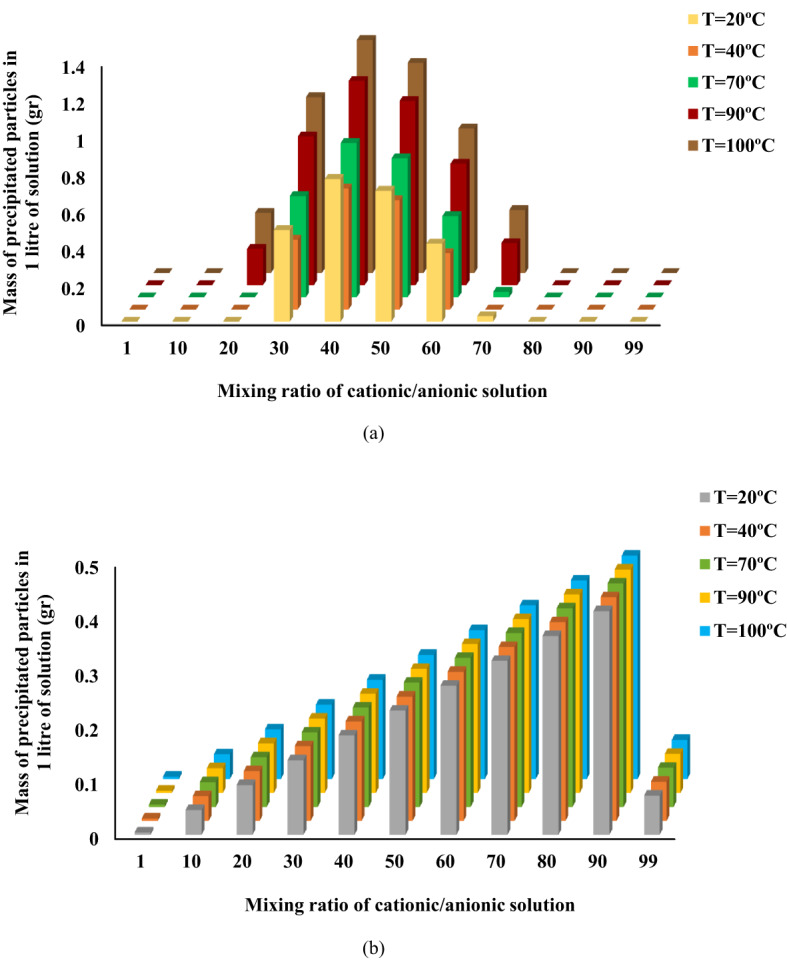
Impact of temperature on the mass of precipitation for different mixing ratios for: (**a**) calcium sulfate, and (**b**) barium sulfate particles.

According to Fig. 3, the mass of precipitation is affected by the mixing ratio and temperature. It could be drawn that for both types of sulfate particles, the mass of precipitation was maximized. Figure 3a showed that the critical mixing ratio in obtaining the maximum mass of calcium sulfate precipitation was 40:60% (cationic to anionic), although the stoichiometric molar ratio of anions to cations was 1:1 (Eqs. 1 & 2). On the other hand, the critical mixing ratio of 90:10% (cationic to anionic) was observed for barium sulfate precipitation, according to Fig. 3b. This ratio could be scientifically expected and justified due to the lowness of barium cations in almost lower mixing ratios. In other words, the mixing ratio of 90:10% would provide an optimal condition for the precipitation of barium sulfate by ensuring a sufficient concentration of barium cations relative to the more available sulfate anions.

Moreover, as shown in Fig. 3, temperature profoundly affects the mass of precipitated sulfate particles of both calcium and barium sulfates. This, in turn, helps to determine the critical mixing ratios. In addition, higher ionic strengths, which leads to maximum mass of precipitated particles, would intensify with increased temperature as shown in the Z-axis of Fig. 3.

**Fig. 4:**
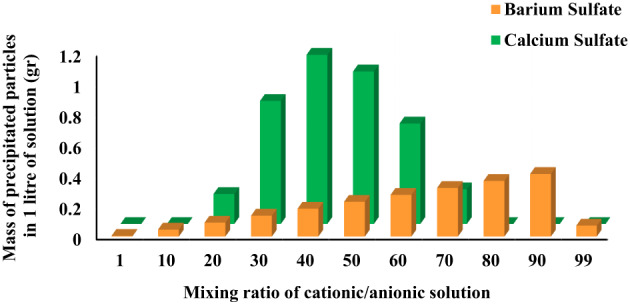
Impact of mixing ratio on the mass of precipitation at a temperature of 90 °C.

To facilitate a direct comparison of the effect of the mixing ratio on the mass of precipitation of sulfate particles at 90 °C, Fig. 4 was hence presented. These findings underline the role of critical mixing ratio in the conducted high temperature static tests. It means that the mixing ratio which resulted in the maximum amounts of precipitated particles at 90 °C was considered the critical mixing ratio for the basis of experimental procedure, as stated previously in Sects. 2–2.

### Impact of mixing ratio on deposition

**Fig. 5:**
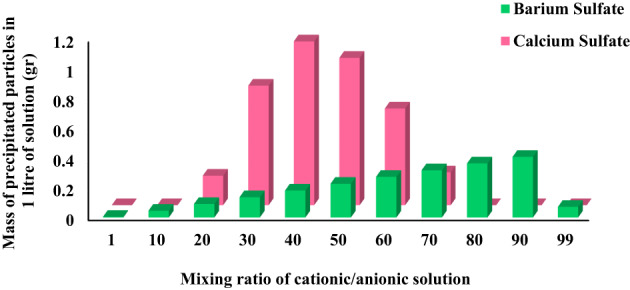
Impact of mixing ratio on the mass of deposition at a temperature of 90 °C in the presence of 5 gr dolomite.

Figure 5 illustrates that the critical mixing ratios for the deposition of calcium and barium sulfate at 90 °C in the presence of a dolomite rock surface closely matched the precipitation behavior observed in the absence of rock. Specifically, the mixing ratios of 40:60 for calcium sulfate and 90:10 for barium sulfate were again identified as the conditions yielding the highest deposited mass.

A comparative analysis of Figs. 4 and 5 reveals that the total deposited masses in the presence of dolomite are not significantly different from the precipitation masses obtained in its absence. This finding strongly suggests that the chemical nature of the dolomite rock surface does not substantially influence the deposition process of these sulfate scales under the tested conditions. It is also worth to highlight that precipitation does not automatically translate into deposition, and yet the presence of dolomite may fail to suppress the deposition in a meaningful way. Therefore, it may be inferred that, at least under the simulation conditions of this study, dolomite rock plays a negligible role in altering the deposition of calcium and barium sulfates since PHREEQC does not inherently account for physical parameters such as surface roughness, purity, or site heterogeneity. However, more investigation on the impact of dolomite rock would be required before a firm conclusion can be drawn.

### Simulated results: Aspen plus

**Fig. 6:**
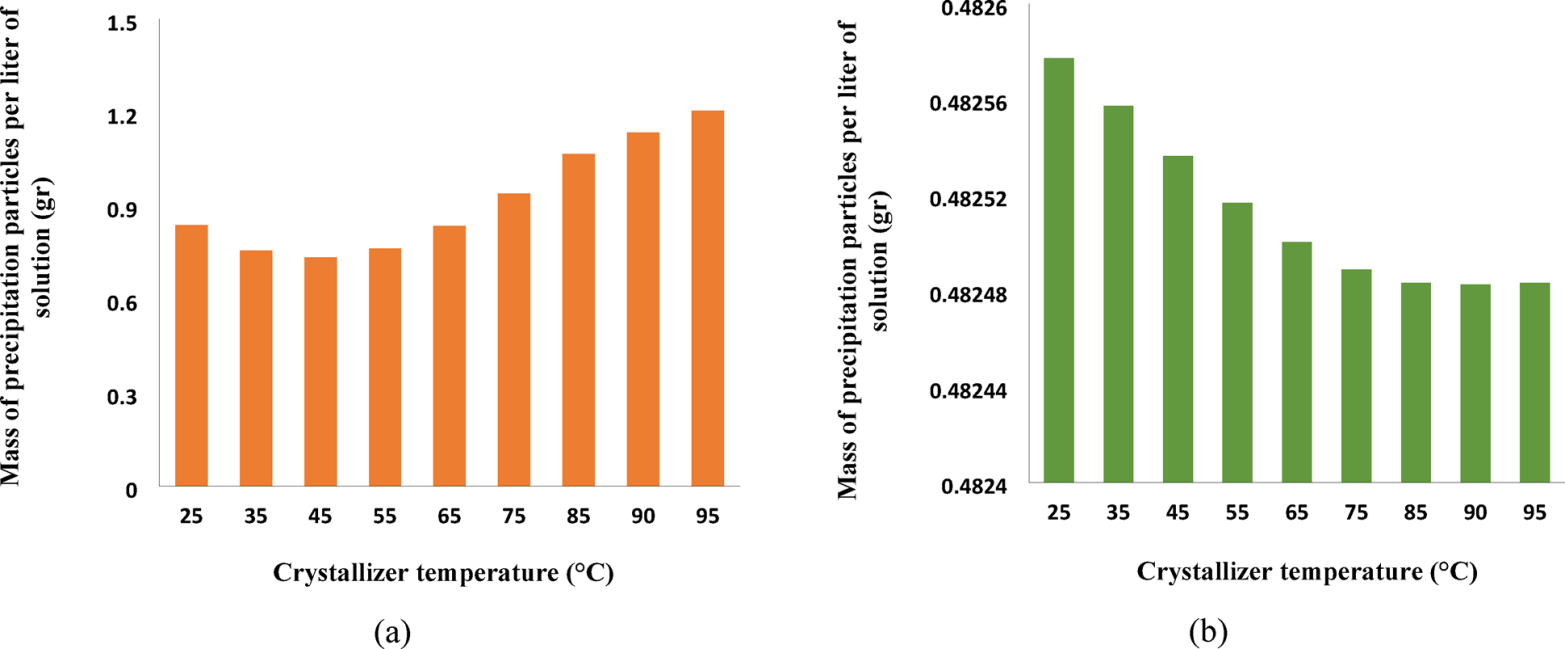
Impact of temperature on the mass of precipitation: (**a**) calcium sulfate, (**b**) barium sulfate.

As prior said, this study, in parallel with PHREEQC, has specifically aimed to employ Aspen plus simulator to contemplate the temperature changes’ impact on the sulfate precipitation. Accordingly, Fig. 6 demonstrates the variation of the precipitated mass versus crystallizer temperature.

Figure 6a indicated a descending then followed by an ascending trend of the mass of calcium sulfate precipitated particles with increased temperature. This behavior may imply that the solubility of calcium sulfate decreases at temperatures above 40 °C, causing an increase in the precipitate mass, as affirmed by previous studies^49,50^. In other words, the literature states that the precipitation rate decreases at the temperature range of 25–45 °C. However, at T > 45 °C, the precipitate mass increases^8,39^ which was observed in this study, as shown in Fig. 6a.

On the other hand, Fig. 6b shows how the barium sulfate precipitated mass varies as a function of the crystallizer temperature. Unlike calcium sulfate, the precipitated mass of barium sulfate decreases with increase in temperature which would be attributed to the barium sulfate solubility increase at higher temperatures^51–53^.

Conclusively, Figs. 3 and 6 could sufficiently validate each other showing a consistent trend of precipitated mass changes versus temperature changes for almost all mixing ratios. However, the amounts may differ from each other which may be related to the simulators’ thermodynamically and kinematically approaches of PHREEQC and Aspen Plus, respectively.

### Experimental results

#### Impact of si’s concentration on precipitation reduction

The performance of folic acid as a green scale inhibitor to combat calcium and barium sulfate precipitation was experimentally examined. Three various concentrations of 500, 100, and 2000 mg/L of folic acid were employed at a constant temperature of 90 °C and atmospheric pressure. Accordingly, to quantitatively evaluate the inhibitory effect of folic acid, the IE% of precipitation was calculated using Eqs. 3 and 4. It was aimed to determine the optimal concentration of folic acid which resulted in the most reduction of precipitation.

**Fig. 7:**
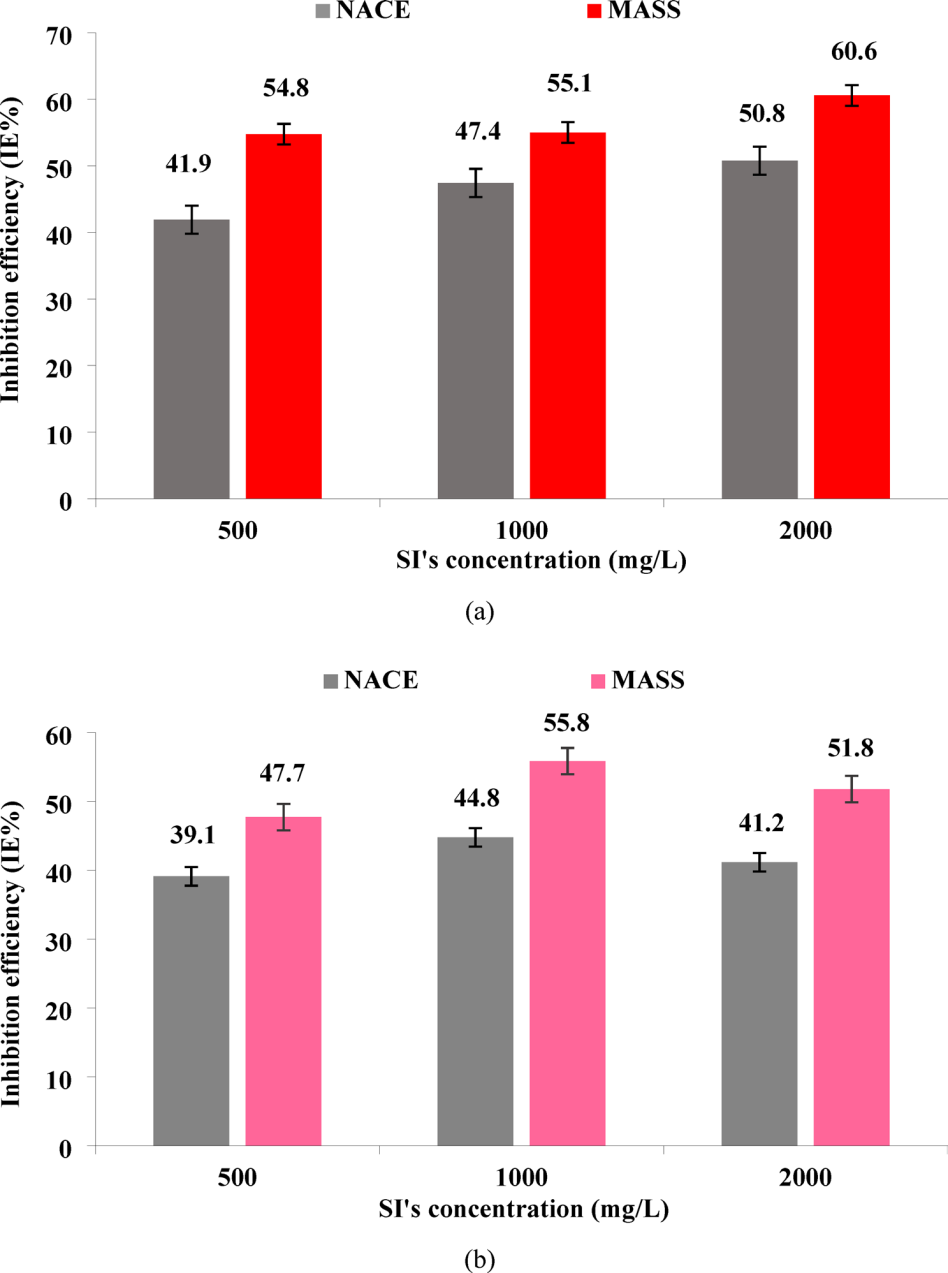
Inhibition efficiency (NACE standard (Eq. 3) and Mass changes (Eq. 4)) of folic acid as a function of SI’s.

Subsequently, Fig. 7a demonstrated that increasing the SI’s concentration was consistently associated with an increase in the IE%. The remarkable amount of calculated inhibition efficiency was reported at 50.8% and 60.6% based on Eqs. 3 and 4, respectively. It was particularly related to the concentration of 2000 mg/L of folic acid which may confirm the effectiveness of folic acid in controlling the calcium sulfate precipitation at high concentrations.

concentration for (a) calcium sulfate, (b) barium sulfate’s precipitation (± 5%).

On the other hand, a dissimilar pattern was illustrated for barium sulfate precipitate as shown in Fig. 7b. According to both Eqs. 3 and 4, the most significant value of folic acid IE% (55.8% and 44.8%) was obtained from 1000 mg/L of the green SI. It means that higher concentration did not lead to further improvement, resulting in lower IE%. Two factors would probably be responsible for this propensity: (i) occupation of utter active sites on the barium sulfate crystals leading to the saturation of the inhibitory performance of folic acid^24,26,54–59^, and (ii) modification of barium sulfate crystals with smaller dispersed sizes with higher specific surface area^57,60^. It will be discussed in detail in Sects. 4-3-3 to explore the inhibition mechanism.

Thus, the optimal folic acid concentrations that showed the highest precipitation inhibition were chosen to further examine the rock surface impact on the SI’s performance. These concentrations of 2000 and 1000 mg/L for calcium and barium sulfate hence were respectively included in deposition studies.

It is worth mentioning that the differences in inhibition efficiency (%IE) calculated by Eq. 3 and Eq. 4 are fundamentally due to the distinct nature of the measurement methods. Specifically, the lower result from the concentration-based method (NACE), as shown in Fig. 7, is actually a favorable trend, as it confirms the inhibitor successfully stabilized ions in solution.

**Table 3:** Commercial SI effectiveness in combat of calcium and barium sulfate particles’ precipitation.

IE% (± 5%) precipitation type	Based on Eq. 3	Based on Eq. 4
Calcium sulfate (15 ml/L of Commercial)	55.7	80.7
Barium Sulfate (15 ml/L of Commercial)	52.1	74.6

To comparatively evaluate the performance of folic acid against a commercial phosphonate-based SI, a similar experimental procedure was applied to investigate the precipitation mass of calcium and barium sulfate particles. The commercial SI showed more reasonable performance compared with folic acid according to Table 3. In accordance with Eqs. 3 and 4, the calculated IE% were estimated at 80.7% and 55.7% for calcium sulfate, and 74.6% and 52.1% for barium sulfate, respectively. This superior phosphonate inhibitory performance would be attributed to the formation of stable phosphonate-cation complexes to thwart nucleation through the threshold inhibition mechanism.

### Impact of SI on deposition reduction

In the interest of the simultaneously investigating the rock presence and folic acid performance to inhibit calcium and barium sulfate deposition, 5 g of the crashed dolomite rock were placed in the recent aqueous system at 90 °C. The deposition tests were performed using the optimal concentration of folic acid, which was estimated in the previous precipitation experiments (Sect. "Impact of SI’s concentration on precipitation reduction") followed by comparing with the blank solution without inhibitor.

According to Table 4, folic acid could effectively hinder the sulfate particles’ deposition in the presence of dolomite rock surface causing a notable reduction of the deposited mass and final cations’ concentration regarded to the calculated IE% by two mentioned equations (Eqs. 3 & 4). The findings would indicate a significant impact of the SI on the reduction of sulfate particles’ deposition in the presence of the rock surface as the reservoir rock representer compared to the conjugated precipitation IE% presented in Fig. 7. It could be drawn that the IE% of the calcium and barium precipitation through 2000 and 1000 mg/L of folic acid concentration were 50.8 and 44.8 estimated based on Eq. 3. While, 60.6% and 55.8% were calculated in accordance with Eq. 4 for calcium and barium sulfate precipitation, respectively (Fig. 7).

Nonetheless, the incorporation of dolomite rock into the system is likely to introduce additional complexity to the scale deposition process by serving as a potentially active surface in particle–solution–rock interactions^61,62^. This suggests that, beyond the chemical parameters such as the saturation index (SI), the presence of solid surfaces may lead to more intricate behavior in mineral scaling systems. Accordingly, a thorough evaluation of SI-related phenomena necessitates the simultaneous investigation of inhibition efficiency and the associated inhibition mechanisms to fully understand the performance of the scale inhibitor under such conditions^36,39^.

**Table 4:** Folic acid and commercial SI effectiveness for mitigation of calcium and barium sulfate deposition.

IE% (± 5%) Deposition type	Based on Eq. 3	Based on Eq. 4
Calcium sulfate (2000 mg/L of folic acid)	53.4	62.7
Barium Sulfate (1000 mg/L of folic acid)	47.2	57.8
Calcium sulfate (15 ml/L of Commercial)	32.7	59.3
Barium Sulfate (15 ml/L of Commercial)	30.2	43.4

A comparative evaluation of the inhibition of inorganic sulfate deposition was additionally done through the commercial SI. Accordingly, Table 4 presented that folic acid could efficiently perform compared to the commercial SI. Table 4 states that the ionic IE% (Eq. 3) of the commercial inhibitor was 32.7% and 30.2% for calcium and barium sulfate, respectively. Moreover, the IE% based on the mass difference (Eq. 4) were also determined respectively 59.3% and 43.4% for calcium and barium sulfate.

In comparison to Tables 3 and 4, the commercial SI performed weaker in sulfate deposition than precipitation. This can be attributed to the SI’s inhibition mechanism i.e. the threshold inhibition to retard the nucleation step of crystallization. On the other hand, folic acid showed a better performance in the deposition phenomenon according to Table 4. It could therefore be inferred that the commercial inhibitor had a stronger performance to retard the precipitation of sulfate particles, while folic acid was more effective in the deposition phenomenon.

It could be argued that folic acid provides a comparable performance to that of commercial SI. Despite its lower efficiency in the precipitation phenomenon, its biocompatibility and effectiveness in deposition mitigation would make it a more sustainable alternative that warrants greater attention in the oil industry, especially where environmental considerations are prioritized^34^.

### Inhibition mechanisms

Equipment analyses of SEM and EDX were employed to identify the inhibition mechanism of folic acid while exposed to calcium and barium sulfate particles in this study. Accordingly, each collected particle sample of different folic acid concentrations that had been used in the precipitation experiments was scrutinized through SEM and EDX to verify the morphology and elemental composition, respectively. However, it was dominantly aimed at elucidating the SI’s inhibition mechanism at the critical mixing ratio condition that induced notable precipitation.

**Fig. 8:**
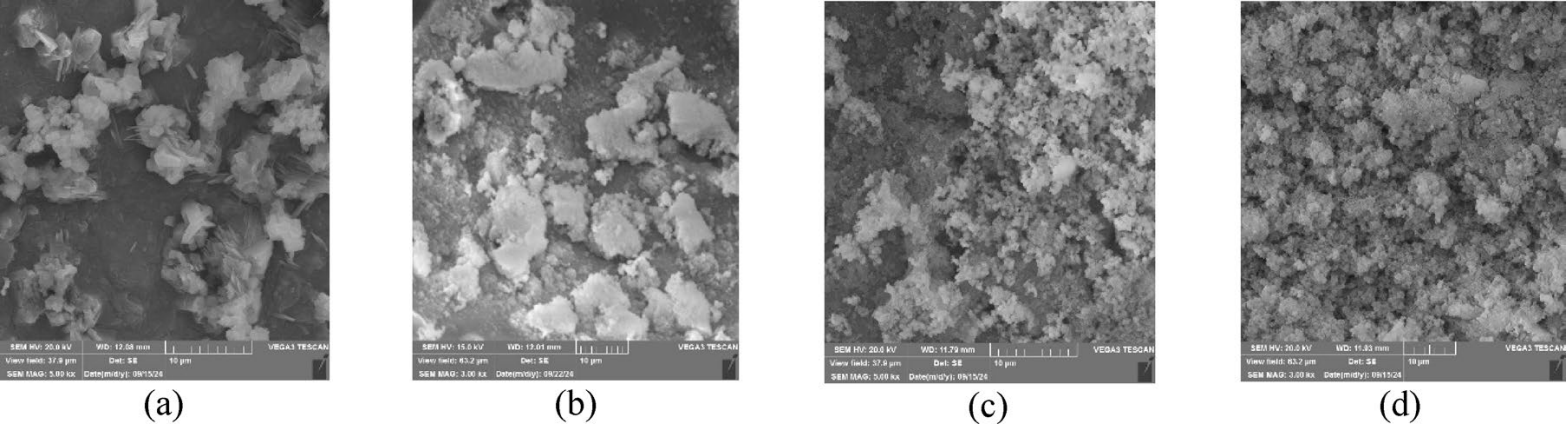
Morphology of precipitated calcium sulfate particles from SEM analysis: (**a**) solution without SI, (**b**) solution with 500 mg/L of the SI, (**c**) solution with 1000 mg/L of the SI, (**d**) solution with 2000 mg/L of the green SI.

**Fig. 9:**
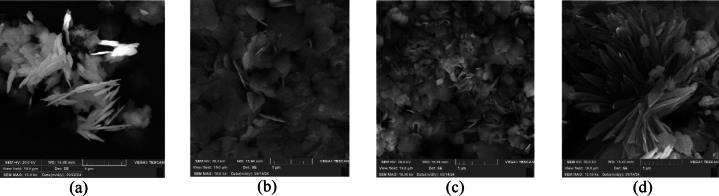
Morphology of precipitated barium sulfate particles from SEM analysis: (a) solution without SI, (b) solution with 500 mg/L of the SI, (c) solution with 1000 mg/L of the SI, (d) solution with 2000 mg/L of the green SI.

Figures 8 and 9 illustrated the SEM images respectively taken from the precipitated calcium and barium sulfate crystals in the presence and absence of the green scale inhibitor of folic acid. Figure 8a hence showed that calcium sulfate crystals are found to be mostly needle-like in the absence of folic acid. While barium sulfate crystals are formed as dense and regular flakes according to Fig. 9a.

On the other hand, in the presence of the green inhibitor folic acid, calcium sulfate crystals retained coarser, more porous, and dispersed structures (Fig. 8b–d). It was intensified by increasing the inhibitor’s concentration as expected. Because the presence of the carboxylic acid functional groups in folic acid would cause more dispersion and disorder of the crystals’ structure by complexing of calcium cations^34,35^. Furthermore, Figs. 9b–d displayed that the barium sulfate crystals acquired a more rounded, porous, and dispersed morphology which mitigated the formation of the hard and cohesive precipitates. It could be hence drawn that the green SI of folic acid would thwart both sulfate precipitations through the crystal modification as the inhibition mechanism.

‎‬‬‬‬‬.

Table 5 presents the EDX analysis results based on the atomic percent of each element. It could be concluded that the atomic percentages of both calcium and barium were decreased in the presence of folic acid. A consistent finding was observed that showed a decrease of the cation’s atomic percentage with the SI’s concentration. It would again be confirmed that folic acid can inhibit sulfate precipitation followed by deposition by disrupting the formation of dense crystals through complexing and dispersing cations. In conclusion, the consistency between SEM and EDX analysis results and the estimated inhibition efficiencies would strongly confirm the effectiveness of folic acid in mitigating the calcium and barium sulfate crystals formation.

**Table 5:** EDX analysis of the precipitated calcium and barium sulfate particles in the presence of the green SI of folic acid.

Sample		weight%					
		*Ca*	*Ba*	*O*	*Na*	*S*	*Cl*
CaSO_4_	Precipitated particles without SI	20.20	-	67.50	2.90	8.50	0.90
	Precipitated particles with 500 mg/L of folic acid	10.70	-	79.50	2.30	7.00	0.50
	Precipitated particles with 1000 mg/L of folic acid	8.40	-	83.00	2.15	6.20	0.43
	Precipitated particles with 2000 mg/L of folic acid	7.10	-	86.50	2.10	3.90	0.40
BaSO_4_	Precipitated particles without SI	-	91.45	6.00	0.50	1.60	0.45
	Precipitated particles with 500 mg/L of folic acid	-	81.50	15.10	1.50	1.40	0.50
	Precipitated particles with 1000 mg/L of folic acid	-	56.60	30.00	6.60	0.80	6.00
	Precipitated particles with 2000 mg/L of folic acid	-	65.80	25.00	4.50	1.20	3.50

**Fig. 10:**
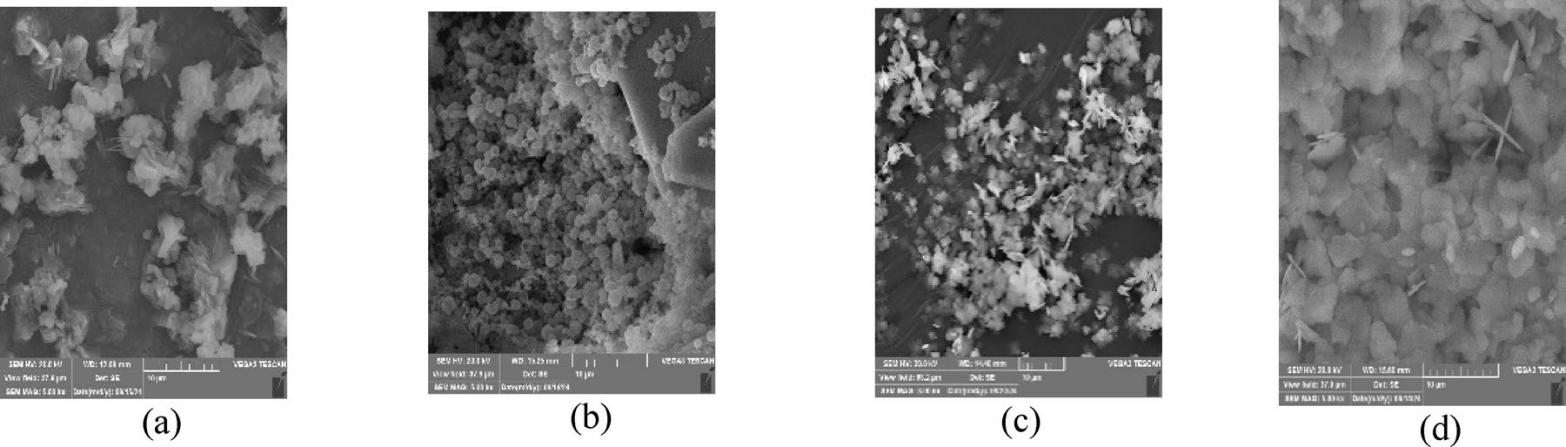
Morphology of precipitated particles from SEM analysis: (a) solution without SI of calcium sulfate particles, (b) solution with the commercial SI of calcium sulfate particles, (c) solution without SI of barium sulfate particles, (d) solution with the commercial SI of barium sulfate particles.

As previously mentioned, the commercial SI was also scrutinized to compare folic acid. Consequently, SEM images were additionally provided in Fig. 10. It distinguished the morphology of the calcium and barium sulfates’ crystals in the presence of the commercial inhibitor in comparison without any SI. According to Fig. 10, as mentioned earlier (Sects. 4-3-1), the commercial SI seems to prohibit the nucleation of the sulfate crystals. This means that it could hinder the initial crystal structures through the threshold inhibition mechanism.

Moreover, EDX analysis results which were presented in Table 6 confirm that less amounts of calcium and barium concentrations, in terms of atomic%, were present in the samples containing the commercial SI, compared to those without the commercial SI. According to similar observations and findings in SEM images, it is evident that the threshold effect is the dominant mechanism of inhibition for the commercial inhibitor. Moreover, the presence of phosphorus in the crystal structure can be considered an indicator for the presence of commercial inhibitor.

**Table 6:** EDX analysis of the precipitated calcium and barium sulfate particles in the presence of the commercial SI.

Sample	weight%						
	Ca	Ba	O	Na	Cl	S	*P*
Calcium sulfate precipitated particles without SI	20.20	-	67.50	2.90	0.90	8.50	-
Calcium sulfate precipitated particles with the commercial SI	5.30	-	63.04	7.88	2.07	4.50	16.20
Barium sulfate precipitated particles without SI	-	91.45	6.00	0.50	0.45	1.60	-
Barium sulfate precipitated particles with the commercial SI	-	70.33	4.42	2.81	1.66	1.05	19.73

## Conclusions

In this study, the comprehensive study of calcium and barium sulfates’ precipitation and deposition in the presence of a green scale inhibitor was done from both points of view, i.e., experimental and simulation. It was primarily intended to determine the impact of mixing ratios of the incompatible solutions. i.e., cationic and anionic ones on the precipitation phenomenon at 90 °C using the PHREEQC simulation tool. The simulated results were hence employed to evaluate the inhibition efficiency of folic acid as the investigated green SI via the high-temperature standard static tests. Moreover, to gain a deeper understanding of the inhibition mechanisms, SEM and EDX analyses were respectively performed via the recognition of the crystals’ morphology and elemental composition (weight%) of the precipitated particles. In the end, the performance of a commercial phosphonate-based SI was also evaluated similarly for comparison with the studied green SI to emphasize the comparable efficiency and potential of folic acid in comparison with the commercial one. As an innovative approach, Aspen Plus software was additionally employed to validate the PHREEQC and experimental results that were conjugated to the precipitation without any SI. Consequently, the key outcomes would be stated as the bullet points in the following:

- PHREEQC simulated results showed that the mixing ratio of cation and anion solutions could play a decisive role in the amount of precipitation. The mixing ratios (cationic to anionic solutions) of 40 to 60 for calcium sulfate and 90 to 10 for barium sulfate were respectively concluded as the critical mixing ratios leading to the maximum amount of precipitation at 90 °C.
- Aspen Plus simulation indicated that increasing temperature would lead to an increase in calcium sulfate precipitation, while this effect would be manifested in the form of a decrease in barium sulfate precipitation as expected according to the difference of their solubility trend versus temperature changes.
- Experimental results demonstrated that the optimal concentrations (minimum amount of precipitation) of folic acid at 90 °C were determined to be 2000 and 1000 mg/L for calcium and barium sulfate, respectively.
- The active carboxylic acid functional groups of folic acid’s chemical structure could effectively control the crystal growth through the cation complexing, called the crystal modification inhibition mechanism base on SEM/EDX analysis. However, it is recommended to perform quantitative image analyses like crystal size distribution and X-Ray Diffraction (XRD) analysis, which would help to figure out the mechanism of inhibition more reliably.
- The commercial phosphonate-based SI could effectively inhibit the precipitated sulfate particles through the threshold inhibition mechanism. However, it had struggled with a challenge in inhibiting the deposition due to the presence of nucleation surfaces provided by the dolomite rock.
- Folic acid revealed stronger performance than the commercial SI mitigating the sulfate deposition in the presence of the dolomite rock surface.

## Data Availability

The datasets generated and/or analysed during the current study are available from the corresponding author on reasonable request.
